# Inflammasome activation and regulation: toward a better understanding of complex mechanisms

**DOI:** 10.1038/s41421-020-0167-x

**Published:** 2020-06-09

**Authors:** Danping Zheng, Timur Liwinski, Eran Elinav

**Affiliations:** 1 Immunology Department, Weizmann Institute of Science, Rehovot, 7610001 Israel; 2 Department of Gastroenterology, The First Affiliated Hospital, Sun Yat-sen University, Guangzhou, China; 3 1st Department of Medicine, University Medical Center Hamburg-Eppendorf, Hamburg, Germany; 4 Cancer-Microbiome Division Deutsches Krebsforschungszentrum (DKFZ), Neuenheimer Feld 280, 69120 Heidelberg, Germany

**Keywords:** Cell signalling, NOD-like receptors

## Abstract

Inflammasomes are cytoplasmic multiprotein complexes comprising a sensor protein, inflammatory caspases, and in some but not all cases an adapter protein connecting the two. They can be activated by a repertoire of endogenous and exogenous stimuli, leading to enzymatic activation of canonical caspase-1, noncanonical caspase-11 (or the equivalent caspase-4 and caspase-5 in humans) or caspase-8, resulting in secretion of IL-1β and IL-18, as well as apoptotic and pyroptotic cell death. Appropriate inflammasome activation is vital for the host to cope with foreign pathogens or tissue damage, while aberrant inflammasome activation can cause uncontrolled tissue responses that may contribute to various diseases, including autoinflammatory disorders, cardiometabolic diseases, cancer and neurodegenerative diseases. Therefore, it is imperative to maintain a fine balance between inflammasome activation and inhibition, which requires a fine-tuned regulation of inflammasome assembly and effector function. Recently, a growing body of studies have been focusing on delineating the structural and molecular mechanisms underlying the regulation of inflammasome signaling. In the present review, we summarize the most recent advances and remaining challenges in understanding the ordered inflammasome assembly and activation upon sensing of diverse stimuli, as well as the tight regulations of these processes. Furthermore, we review recent progress and challenges in translating inflammasome research into therapeutic tools, aimed at modifying inflammasome-regulated human diseases.

## Introduction

Inflammation is a vital physiological response triggered by noxious agents in all metazoan organisms. Virtually any challenge to the body’s homeostasis may elicit an inflammatory response at the local or systemic levels^1^. The innate immune system’s task is to generate a protective response against signals of danger including both pathogenic microorganisms and sterile incursions such as trauma, cancer, ischemia, and metabolic perturbations. Technically, factors eliciting an innate inflammatory response can be classified as pathogen-associated molecular patterns (PAMPs), conserved compounds of infectious agents, and damage-associated molecular patterns (DAMPs), which are signals of host cellular distress. PAMPs and DAMPs are sensed by an increasingly appreciated variety of pattern recognition receptors (PRRs) and cells of innate and adaptive immunity^2,3^.

Upon encounter of a pathogenic agent or tissue injury, the innate immune system is challenged to integrate a wealth of signals to initiate a proper response. In addition to Toll-like receptors (TLR)^4^, Lectin receptors^5^, RIG-I-like receptors^6^, and oligoadenylate synthase (OAS)-like receptor^7^, inflammasomes emerged in the last decade to constitute fundamental processing units contributing to PAMP and DAMP sensing, which actively participate in integration of their downstream signaling^3,8^. The term “inflammasome” has originally been coined by Martinon et al.^8^ in a seminal report in 2002 that described the assembly of these supramolecular structures in the cytoplasm of activated immune cells, thereby leading to proteolytic activation of proinflammatory caspases, which drives subsequent systemic immune responses and inflammation. Importantly, while inflammasome signaling has been shown to be critical to host defense, the elicited immune response needs to be tightly regulated in order to limit collateral damage to the host. This implies that appropriate regulation of inflammasomes is intrinsic to the control circuit of the associated inflammatory processes.

Several cytoplasmic PRRs are able to assemble into an inflammasome complex and are classified by their protein domain structures. For example, the NBD leucine-rich repeat-containing receptor (NLR) family implicates subfamilies distinguishable by their N-terminal effector domains. There are four recognizable NLR N-terminal domains: the acidic transactivation domain, pyrin domain, caspase recruitment domain (CARD), and baculoviral inhibitory repeat (BIR)-like domains^9^. Another class of inflammasome assembling PRRs is represented by PYHIN protein family members, such as absent in melanoma 2 (AIM2), which contain HIN200 and pyrin domains^10^. Inflammasome-assembling PRRs are expressed in many cell types, including macrophages, dendritic cells (DCs), neutrophils, and epithelial cells^11^. The final common pathway of inflammasome signaling is inflammatory caspase-activation. This task is achieved by the assembly of a hetero-oligomeric complex based on a scaffold protein, such as NOD-like receptor-pyrin-containing proteins (NLRP) or AIM2 protein, and in some inflammasomes the additional recruitment of adapter and effector partners, such as apoptosis-associated speck-like protein containing a CARD (ASC)^12^. The activation of caspases results in the proteolytic activation of the proinflammatory cytokines interleukin-1β (IL-1β) and/or interleukin-18 (IL-18). In particular, IL-1β is considered a gatekeeper cytokine which is critically involved in many events related to activation and regulation of inflammation^13^.

Considering the potency of the inflammasome-dependent immune responses, it is not surprising that dysregulated inflammasome activity is associated with a number of inflammatory disorders or of multi-factorial diseases involving an inflammatory component, including autoinflammatory disorders, cardiometabolic diseases, infection, cancer and neurological disorders^12,14–16^. Therefore, regulation of inflammasome activity and therapeutic interventions targeting structures related to inflammasome signaling constitute promising areas of basic and translational research. As it is impossible for a single review to fully cover the large and rapidly growing body of high-quality research in inflammasome biology, we aim to provide an introduction to key concepts and an update on recent evidence highlighting new aspects of inflammasome signaling regulation and its implications in health and disease, while referring to reviews dedicated to more specific features of inflammasome research throughout the text.

## Inflammasome activation and assembly

**Fig. 1:**
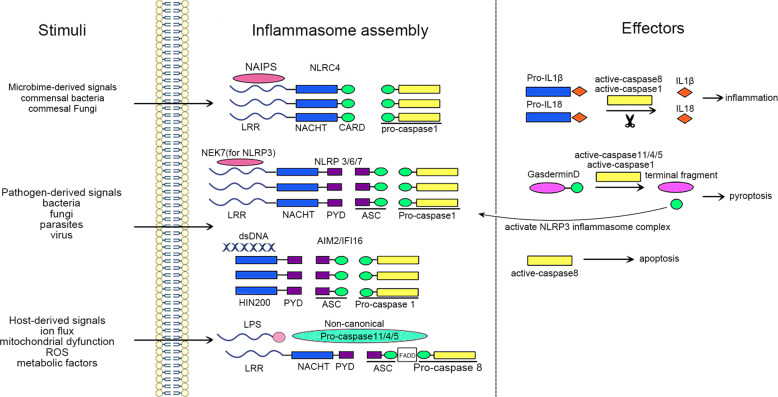
Pattern diagram for inflammasome assembly and activation. Inflammasomes can be activated by a multitude of infectious and sterile stimuli, including microbiome-derived signals (e.g., bacteria, fungi, parasites, and viruses) and host-derived signals (e.g., ion flux, mitochondrial dysfunction, ROS, and metabolic factors). Upon activation, the inflammasome sensors NAIP/NLRC4, NLRP3/6/7, AIM2/IHI16 initiate the canonical inflammasome assembly by recruiting and forming pro-caspase-1 filaments, with or without the ASC adapter. The assembly of non-canonical inflammasomes involves the pro-caspase-11 (−4/5 in humans) or pro-caspase-8. Consequently, the active caspase-1 or caspase-8 leads to the maturation and secretion of inflammatory cytokines IL-1β and IL-18. The active capsase-1 or caspase-11/4/5 triggers the cleavage of GSDMD, which can either cause pyroptosis or activate the NLRP3 inflammasome complex. In addition, the active caspase-8 mediates another effector function, apoptosis.

As an important arm of innate immunity, one of the most outstanding functions of inflammasomes is to detect and sense a variety of endogenous or exogenous, sterile or infectious stimuli that are encountered within the cell, and to induce cellular responses and effector mechanisms. The assembly of the inflammasome platform is a critical and well-organized process involving several core parts: the upstream sensors recognizing activating signals, the adapters and the downstream effectors (Fig. 1).

## Pathogen-derived activating signals

A plethora of PAMPs play key roles in initiating activation of various inflammasomes, among which the most well-studied are bacteria-associated signals. Pathogenic activators of the NLRC4 inflammasome are mainly derived from Gram-negative bacteria namely *Salmonella, Legionella, Shigella*, and *Pseudomonas spp*. These bacteria possess flagellin, or a type III (T3SS) or type IV (T4SS) secretion system rod proteins that are recognized by the NAIP proteins, constituting unique binding partners of NLRC4^17^. The murine NLRP1b inflammasome detects *Bacillus anthracis* lethal toxin in the cytoplasm^18^. The NLRP3 inflammasome can be activated by the pore-forming activity of a wide array of Gram-positive and Gram-negative bacteria, including *Staphylococcus aureus*, *Streptococcus pneumoniae*, *enterohemorrhagic Escherichia coli* and others, mainly through triggering potassium (K^+^) eflux^19–21^. NLRP6 inflammasome can sense lipoteichoic acid derived from Gram-positive pathogens like *Listeria monocytogenes*^22^. In human macrophages, the NLRP7 inflammasome recognizes acylated lipopeptides, a microbial cell wall components^23^. Free cytosolic DNA released from a variety of bacteria species, including but not limited to *Francisella novicida*, is required to activate the inflammasome-forming DNA sensor AIM2^24^. Modification and inactivation of the Rho GTPases by bacterial toxins, for example the *Clostridium difficile* cytotoxin TcdB, and *Clostridium botulinum* ADP-ribosylating C3 toxin, are important to activate the Pyrin inflammasome, dependent on their enzymatic activities^25^. In addition, intracellular lipopolysaccharide (LPS) from Gram-negative bacteria is known to be recognized by the mouse caspase-11, or human caspase-4/5, in activating non-canonical inflammasomes^26^. Notably, although the inflammasome activation facilitates host defense against intracellular pathogenic infections, some bacteria have developed effective strategies to dampen or evade inflammasome activation, which has been recently reviewed in detail elsewhere^27^. For example, bacteria such as *Streptococcus spp*. can restrain inflammasome activation by producing hydrogen peroxide, which in turn dampens bacterial clearance from host cells^28^.

Moreover, specific factors from viruses, fungi and parasites have been recently shown to also activate inflammasomes. For example, the NLRP3 inflammasome in macrophages can be activated by a multitude of viruses and viral proteins, such as the hepatitis C virus core protein^29^, severe acute respiratory syndrome coronavirus (SARS-CoV) viroporin^30^, Influenza virus M2^31^, and Encephalomyocarditis virus viroporin 2B^32^. NLRP9b, mainly expressed in intestinal epithelial cells (IECs), is capable of recognizing the short double-stranded RNA of rotavirus through host RNA helicase Dhx9^33^. Major fungal PAMPs such as β-glucan upon *Aspergillus fumigatus* infection^34^, fungal CPG^35^, Candidalysin secreted by *Candida albicans*^36,37^ are direct inducers of inflammasome assembly, mainly for the NLRP3 inflammasome. Moreover, activation of both canonical and noncanonical inflammasomes (mainly involving mouse caspase-11 or human caspase-4/5) by diverse parasitic stimuli, such as Leishmania and its lipophosphoglycan^38,39^ and *Fasciola hepatica*-derived molecule cathepsin L3^40^ have recently been discovered as an important strategy for the restriction and control of parasitic invasion.

The mammalian body is inhabited by a complex community of microorganisms, collectively termed the microbiome. Selective commensal bacteria can not only induce IL-18 secretion in IECs, which is mediated by NLRP6 inflammasome^41^, but also induce IL-1β maturation mediated by NLRP3 inflammasome signaling in intestinal monocytes, which subsequently promotes intestinal inflammation^42^. NLRP3 inflammasome activation induced by commensal bacteria is K^+^ efflux-dependent, but does not depend on bacterial viability or phagocytosis^43^. Sensing of commensal gut fungi through the Card9–Syk signaling axis promotes inflammasome activation and maturation of IL-18, which plays a protective role in colitis and colitis-associated carcinogenesis^44^.

### Host-derived activating signals

In addition to PAMPs, endogenous DAMPs (or host danger signals), which are released upon tissue injury, emerged as another major source for inflammasome activation. For example, NLRP3 inflammasome which is activated by a diverse numbers and structures of exogenous stimuli ranging from particulate matter (such as silica) to bacterial-derived toxins^45^ can also sense other host-derived signals downstream of all these exogenous stimuli. Some of these endogenous signals, including ion efflux, mitochondrial dysfunction and reactive oxygen species (ROS) are well-characterized (see below), while others remain uncharacterized to date. An elegant example of a new host-related activator was demonstrated in a recent study showing that diverse NLRP3 stimuli can trigger the disassembly of trans-Golgi network (TGN), and the dispersed TGN acts, in turn, as a scaffold for NLRP3 recruitment through ionic bonding and subsequent inflammasome assembly and activation^46^.

Cytosolic K^+^ efflux is a common trigger involved in both canonical NLRP3 inflammasome activation^21^ and caspase-11-mediated noncanonical activation^47^. NEK7, a mitosis-related serine-threonine kinase, is essential to regulate NLRP3 oligomerization and activation downstream of K^+^ efflux^48,49^. Intracellular chloride efflux, another event downstream of K^+^ efflux, is a critical upstream event for NLRP3 activation^50^. The role of calcium (Ca^2+^) signaling in activating NLRP3 inflammasome remains debatable. Although some studies showed that inflammasome activation essentially requires elevation of intracellular and extracellular Ca^2+^^51,52^, other studies argued that Ca^2+^ is not strictly required for initiation of NLRP3 signaling^53,54^. The roles and mechanisms of other upstream ion flux disturbances, including Na^+^ and Zn^2+^ in orchestrating NLRP3 inflammasome activation have been extensively reviewed elsewhere^55^. Although it remains to be answered whether these ions directly bind to NLRP3 and trigger downstream processes, such advances provide potential ion-associated targets for modulating NLRP3-driven disorders.

Mitochondrial dysfunction is another emerging elicitor of NLRP3 activation, operating via mitochondrial DNA (mtDNA) release, mitophagy and apoptosis. For example, in aging hematopoietic stem cells, increased mitochondrial stress leads to aberrant NLRP3 inflammasome activation and contributes to characteristic aging-associated defects^56^. Recently, it was found that mtDNA synthesis and oxidized mtDNA release into the cytosol driven by TLR signaling is crucial to prime NLRP3 activation, while inhibition of mtDNA synthesis via TFAM or IRF1 ablation prevents NLRP3 inflammasome activation^57^. However, other studies show that TFAM depletion leads to increased rather than decreased cytosolic mtDNA to activate antiviral immune responses through the cGAS–STING pathway^58^, and that IRF1 is dispensable for the NLRP3 inflammasome activation^59^. Furthermore, cholesterol-dependent mtDNA accumulation in macrophages results in AIM2, but not necessarily NLRP3 inflammasome activation^60^. Cytosolic mtDNA activates the NLRP3 inflammasome and promotes the release of IL-1β and IL-18, while the translocation of mtDNA into cytosol, in turn, requires NLRP3, indicating that NLRP3 might act both upstream and downstream of mtDNA release^61^. On the other hand, damaged mitochondria, stimulated by NLRP3 activators, initiate an intrinsic “NF-κB-p62-mitophagy” pathway through which NF-κB limits NLRP3 inflammasome activation^62^. These results highlight a regulatory loop existing between the NLRP3 inflammasome and mitochondria. In addition, oxidized mtDNA released into the cytosol during apoptosis binds to the NLRP3 inflammasome, highlighting a link between apoptosis and inflammasome activation^63^. However, this conclusion has been challenged by another study showing that genetic deletion of important executioners of mitochondrial apoptosis does not affect the activation of NLRP3 inflammasome^64^. Indeed, besides mtDNA, intrinsic apoptosis resulting from mitochondrial membrane damage in macrophages activates Caspase-3 and -7 to drive IL-1β secretion downstream of both NLRP3 inflammasome and caspase-8 signaling^65^.

Emerging evidence suggests that ROS constitute another central signaling hub among a diversity of NLRP3-related stimuli^45^. ROS induce the binding of thioredoxin-interacting protein to NLRP3, which is essential for NLRP3 inflammasome activation^66^. Although some studies suggest that NLRP3 inflammasome activation may be triggered by ROS generated by an NADPH oxidase^67^, other studies show that activation of the NLRP3 inflammasome is independent upon NADPH oxidase-generating ROS^68^ or its oxidative activity^69^, challenging the proposed indispensable role of ROS in NLRP3 inflammasome activation. Of note, ROS generation is frequently accompanied by mitochondrial dysfunction and ion efflux. It would be worth investigating the interplay among these triggers in contributing to inflammasome activation.

Growing evidence suggests that dysregulated metabolic factors may constitute novel stimuli of inflammasome activation. These include, as an example, alterations in sterol biosynthesis^70^ and glycolysis metabolism^71^. Cholesterol overload leads to activation of the AIM2 inflammasome through impairing mitochondrial metabolism and eliciting mtDNA release^60^. Increased choline uptake and metabolism in macrophages keeps the NLRP3 inflammasome in an active state by maintaining functional mitochondria^72^. In the central nervous system, cholesterol accumulation in myelin debris in aged mice results in NLRP3 inflammasome activation, which hampers remyelination and nerve repair^73^. Collectively, the discovery of multiple metabolic activators of inflammasomes necessitates the investigation of the underlying mechanisms by which metabolic dysfunction impacts on inflammasome biology.

### Assembly of inflammasomes

Upon activation, assembly of inflammasomes requires interactions between the inflammasome sensor and inflammatory caspase-1 or noncanonical caspase-11, with or without co-binding of the adapter protein ASC. With the help of advanced techniques such as cryo-electron microscopy, rapidly growing evidence begins to disentangle the structural mechanisms of inflammasome assembly.

#### NLRC4 inflammasome

The interaction between the nucleotide-binding domain (NBD) and winged-helix domain (WHD) is essential to keep NLRC4 in an auto-inhibited state^74^. Studies using a purified PrgJ–NAIP2–NLRC4 inflammasome revealed that one single PrgJ-bound NAIP2 molecule with a catalytic surface is sufficient to activate NLRC4 by matching its oligomerization surface, which comprises a large portion of NBD and a small part of LRR. Activated NLRC4 undergoes substantial structural reorganization, interacts, and activates another quiescent NLRC4 molecule in a self-propagating approach to ultimately form a 10- to 12-spoke wheel- or disk-like architecture^75,76^. Similarly, studies on the assembled flagellin–NAIP5–NLRC4 inflammasome showed that the conserved regions of the flagellin ligand recognize multiple domains of the NAIP5 molecule, resulting in NAIP5 activation^77^ and mediating progressive NLRC4 oligomerization^78^. More importantly, the extreme C-terminal side of different bacterial flagellins forms a key structural epitope for their specific detection by NAIP5, potentially explaining why different bacteria possess different potency in inducing the NLRC4 inflammasome^79^. How different NAIPs precisely interact with their respective ligands provides an intriguing question for further investigations.

#### NLRP3 inflammasome

Unlike NLRC4, the assembly of the NLRP3 inflammasome requires the presence of NEK7, a serine and threonine kinase that is critically involved in mitotic cell cycle progression^48,80^. NEK7 directly binds to the LRR domain of NLRP3 to promote inflammasome assembly during cell interphase^49^. A recent cryo-electron microscopic study focusing on the human NLRP3-NEK7 complex found that NEK7 is indispensable to bridge the gaps between adjacent NLRP3 subunits and mediate NLRP3 oligomerization, thereby clarifying the structural basis of NEK7-mediated NLRP3 inflammasome activation^81^. Despite these advances, it remains to be investigated whether NEK7 serves as a common sensor for multiple stimuli-induced NLRP3 activation states, and whether NEK7 is sufficient to trigger a nucleated oligomerization.

#### Other NLRP inflammasomes

In vitro reconstitution of the human NLRP1 inflammasome by purified recombinant proteins has characterized that NLRP1 oligomerization can directly recruit caspase-1 via its CARD domain, and can be further enhanced by binding to ASC via the pyrin domain (PYD)^82^. Crystal structure analysis suggests that interaction between human NLRP1 and procaspase-1 CARDs is potentially mediated by electrostatic attractions^83^. The role of enzymatic cleavage of murine NLRP1b in its activation is discussed in detail below. Another NLRP subfamily member, NLRP6, is capable of forming filamentous structures though the PYD by self-assembly. Remarkable conformational changes following this step, enable subsequent recruitment of the ASC adapter through PYD–PYD interaction, while the NBD of NLRP6 features a synergistic role in enhancing the assembly process^84^. NLRP7 can self-associate to form an oligomer through NACHT domain interaction upon activation^85^, which essentially requires the ATP-binding and hydrolysis activities of the NLRP7 NBD^86^. In a recent study, a previously uncharacterized member of the NLR family, NLRP9b, has been shown to assemble an inflammasome in the intestine upon murine enteric rotavirus infection^33^. The authors demonstrated that NLRP9b recognizes short dsRNA of rotavirus to form inflammasome complexes with ASC and caspase-1 to promote maturation of IL-18 and GSDMA-induced cell death^33^.

#### AIM2 and IFI16 inflammasomes

Assemblies of the AIM2 and IFI16 inflammasomes are considered different from the aforementioned NLR-dependent inflammasomes, since both the AIM2 and IFI16 sensors specifically recognize cytosolic DNA through their hematopoietic interferon-inducible nuclear (HIN) domain, while lacking the NOD for self-oligomerization. Electrostatic binding of double-stranded DNA backbone to the positively charged HIN domain liberates AIM2 autoinhibition^87,88^. Following this event, the helical assemblies of AIM2^PYD^ generate a polymerization platform to nucleate downstream ASC^PYD^ filaments, underpinning the assembly of an inflammasome^89,90^. Unlike AIM2, the isolated IFI16 HIN domain possesses relatively weaker DNA-binding affinity^87^, which can be strengthened by the presence of a full length IFI16 protein^91^. Furthermore, the non-DNA-binding PYD of IFI16 is necessary for the cooperative assembly of IFI16 filaments on dsDNA^91^.

#### Recruitment of ASC and caspase-1

In CARD-absent NLRs including AIM2, IFI16, NLRP3, NLRP6, and NLRP7, PYD–PYD interactions between NLRs and ASC nucleate the PYD filaments of ASC, which in turn nucleates CARD filaments of caspase-1 through CARD–CARD interactions^92^. This brings monomers of pro-caspase-1 into close proximity and initiates caspase-1 self-cleavage and activation. Another innate immune sensor encoded by *MEFV* gene, pyrin, also forms an inflammasome complex in an ASC-dependent approach^93^. Multiple binding sites on both PYDs of pyrin and ASC identified by spectrometry provide a molecular basis for their interactions^94^. In NLRC4, which contains a CARD, the nucleation and activation of downstream caspase-1 can occur through the homotypic CARD–CARD binding, and this process can be independent of ASC^95^. The orientation of the CARD of NLRC4 and the ability to integrate ASC into the structure is currently unknown and warrants further studies.

#### Noncanonical inflammasome activation

While the final common pathway of canonical inflammasome activation involves recruitment of caspase-1 in response to multiple microbial or danger signals, the newly discovered “noncanonical inflammasome” signals in a caspase-1-independent manner through direct recognition of the cytosolic LPS by the CARDs of caspase-4 and caspase-5 (in humans) and caspase-11 (in mice). This elicits caspase-dimerization and activation, resulting in cleavage of GSDMD^96,97^. Activation and regulation of non-canonical inflammasomes is described in detail below.

### Inflammasome effector functions

#### Inflammasome-mediated maturation of IL-1 family cytokines IL-1β and IL-18

Secretion of the proinflammatory cytokines IL-1β and IL-18, together with induction of pyroptotic cell death (see below), represent the main outcomes of inflammasome activation upon caspase-1 activation^98^. Akin to caspase-1, IL-1β, and IL-18 accumulate in the cytoplasm as inactive precursors (pro-IL-1β and pro-IL-18, respectively). Caspase-1 cleaves pro-IL-1β into a 17 kDa mature fragment^99^ and proIL-18 into a 17.2 kDa mature protein^100^. IL-1β stimulates the release of other cytokines such as IL-6, tumor necrosis factor (TNF)-α and IL-1α, as well as other crucial factors responsible for growth and differentiation of immune cells^13^. Both IL-1α and IL-1β bind to IL-1R1, thereby enabling recruitment of its co-receptor IL-1RAc. Similarly, upon binding of IL-18 to IL-18Rα, the latter heterodimerizes with IL-18Rβ. Approximation of the intracellular TIR domains of the IL-1R or IL-18R complex results in recruitment of MyD88 followed by a cascade of downstream events, which ultimately results in the activation of important signaling proteins and transcription factors, such as NF-κB, regulating inflammation^101^.

#### Pyroptosis

Both canonical inflammasome signaling engaging caspase-1 and noncanonical inflammasome activation recruiting caspase-4, caspase-5 (in humans), and caspase-11 (in mice) elicit an inflammatory type of cell death termed “pyroptosis”^102^. Pyroptosis is a lytic form of programmed cell death in response to sensing of pathogens or host-derived danger signals. It is morphologically distinct from apoptosis and is characterized by cell swelling, membrane rupture, and subsequent release of inflammatory compounds into the extracellular space, such as IL‐1β, IL-6, and IL‐18^103,104^. A single protein from the gasdermin family, gasdermin-D (GSDMD) represents the key pyroptotic substrate of inflammatory caspases^105^. GSDMD is expressed in various cell types, with a particularly high density in intestinal epithelia. Pyroptosis plays a critical role in the clearance of intracellular bacteria, effectively resulting in the clearance of the pathogen’s niches^106^. Studies utilizing engineered bacteria showed that pyroptosis of the host cell does not directly cause bacterial death. Instead, the pathogen remains trapped within the cell remnant which is termed “pore-induced intracellular trap (PIT)”. PITs arrest bacteria during simultaneous recruitment of neutrophils and macrophages, which ultimately results in subsequent killing of the pathogen^107,108^.

#### Apoptosis

Caspase-8-driven apoptosis, also regarded as “secondary pyroptosis”, is increasingly studied as another effector function of inflammasome activation. Studies have confirmed the existence of a caspase-8 dependent apoptotic death pathway activated by AIM2, NLRP3, and NAIP–NLRC4 inflammasomes, which is parallel to, but distinct from the canonical caspase-1 dependent pyroptosis, and is present in various cell types^109–111^. In the absence of caspase-1, these inflammasomes are capable of triggering caspase-8-dependent apoptosis upon sensing of diverse stimuli^112,113^. Importantly the presence of caspase-1 protease activity suppresses activation and induction of caspase-8-mediated apoptosis by the sensor NLRP1b and NLRC4^114^. Procaspase-8 binds to the ASC through the adapter protein FADD, and co-localizes to ASC inflammasome specks, adding another layer of complexity to the inflammasome structure^109,114^. Interestingly, a recent study shows that apoptosis induced by inflammasome stimuli can be initiated by caspase-1 itself, in the absence of GSDMD, through the Bid–caspase-9–caspase-3 axis^115^. The molecular mechanisms regulating inflammasome-induced apoptosis, and the interaction between apoptosis and pyroptosis, remain to be further investigated.

## Regulatory mechanisms of inflammasome activation

In general, in a well-timed and carefully controlled inflammatory response, it is critical to seek the optimal balance between inflammasome activation and inhibition. This depends on complex and highly organized regulatory mechanisms. Due to distinctions in the structures and expression patterns of inflammasome sensors, different inflammasomes are activated and regulated by different mechanisms.

### NLRP1 inflammasome

Unlike other NLR family members, human NLRP1 and murine NLRP1b contains a CARD domain at its C-terminus, and a unique function-to-find domain (FIIND). NLRP1 undergoes autolytic proteolysis within the FIIND, which generates N-terminal and C-terminal fragments that remain in an auto-inhibited state, while NLRP1 inflammasome activation can be blocked by abolishing FIIND auto-processing activity^18,116^. Mouse NLRP1b and its rat paralog activate caspase-1 in response to a lethal toxin secreted by *B. anthracis*^18,117^. Both rodent and human NLRP1 molecules are directly proteolytically cleaved by the anthrax lethal factor, which is necessary and sufficient to activate the NLRP1b inflammasome^118,119^. A “functional degradation” model was recently proposed, providing a universal molecular mechanism for murine NLRP1b inflammasome activation induced by diverse pathogen enzymes. Direct cleavage of NLRP1b by pathogen-encoded proteases like lethal factor or Shigella effector IpaH7.8 induces degradation and instability of the NLRP1b N-terminus, thereby liberating the C-terminal fragment to activate caspase-1^120,121^. The N-terminus degradation of NLRP1b is mediated by the N-end rule ubiquitin ligase UBR2, accompanied with an E2 ubiquitin-conjugating enzyme UBE2O, as revealed by genome-wide CRISPR/Cas9 knockout screens^121,122^. Release of the C-terminal FIIND(UPA)–CARD fragment is required to recruit CASP1 and initiate NLRP1b inflammasome assembly^120^.

NLRP1b also acts as an inflammasome sensor for *L. monocytogenes*^123^ and the intracellular parasite *Toxoplasma gondii*^124^, as well as secondary to depletion of cytosolic ATP^125^. It remains to be investigated how human NLRP1 or murine NLRP1b senses specific pathogens, whether these pathogens also activate NLRP1 via “functional degradation”, and whether ATP depletion acts as a unifying event upstream of NLRP1 or NLRP1b inflammasome activation. Furthermore, the role of the NBD and LRR sites of NLRP1 remains elusive. A previous study showed that the PYD and LRR domains of NLRP1 inhibit its self-organization, while disruption of these two domains by NLRP1 mutation leads to inflammasome activation, and contributes to skin inflammation and skin cancer in humans^126^. This provides a novel mechanism for NLRP1 PYD- and LRR-mediated inflammasome regulation, which is also dependent on the auto-proteolytic cleavage within the FIIND domain. However, whether these domains also contribute to the identification of pathogen-encoded effectors warrants further studies.

Additionally, recent evidence shows that inhibition of the cytosolic serine proteases Dpp8 and Dpp9 activates the murine NLRP1b inflammasome to induce pyroptosis, which is dependent on proteasome activity, but does not require proteolysis^127^. Direct binding of human DPP9 to the FIIND and its catalytic activity synergize in keeping NLRP1 in an auto-inhibitory state^128^, while DPP8/9 inhibitors activate murine NLRP1b through an endogenous proteasomal degradation pathway^122^. The substrates of Dpp8/Dpp9 and the molecular mechanisms for this pathway require further investigation.

### NLRP3 inflammasome

**Fig. 2:**
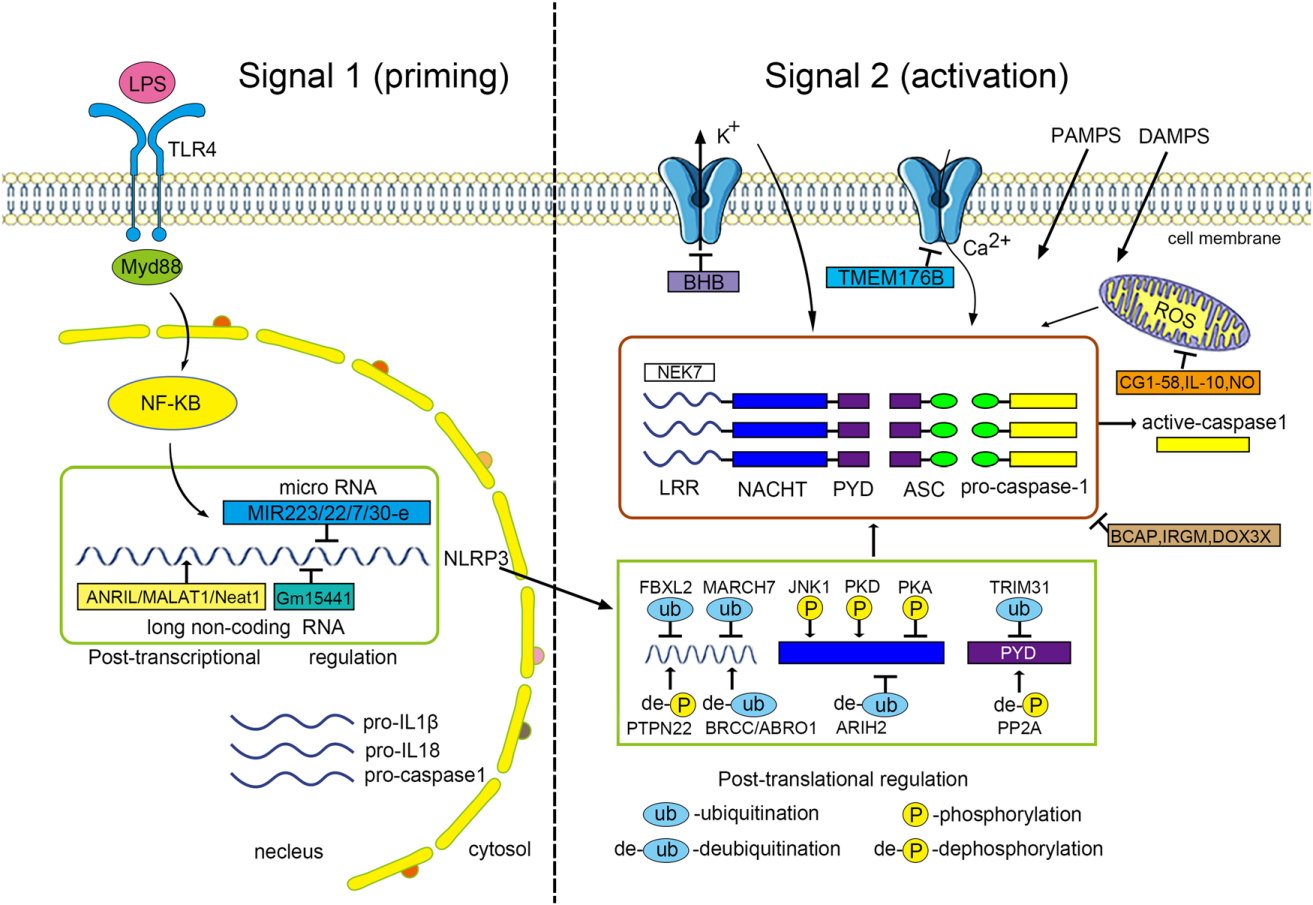
Regulation of the NLRP3 inflammasome activation. Activation of the NLRP3 inflammasome involves both the priming and activation steps. In the priming step (Signal 1), inflammatory triggers such as TLR4 agonist LPS induces the NF-κB-mediated expression of NLRP3, pro-IL-1β and pro-caspase-1. In the activation step, diverse PAMPs and DAMPs trigger the NLRP3 inflammasome assembly though unifying events such as ion flux, mitochondrial dysfunction and ROS formation. Regulation of the NLRP3 inflammasome activation can occur at the post-transcriptional and post-translational levels. MicroRNAs (e.g., MiR-223, MiR-22, MiR-7, and MiR-30e) inhibit the NLRP3 activity by targeting its UTR binding sites, and long non-coding RNAs (e.g., ANRIL, MALAT1, Neat 1, and Gm15441) can either promote or inhibit the inflammasome signaling. The post-translational modifications at different sites and domains of NLRP3 protein include phosphorylation, dephosphorylation, ubiquitination, de-ubiquitination, and S-nitrosylation. Many molecules such as BCAP, IRGM, and DDX3X can interfere the assembly of NLRP3, ASC, and procaspase-1. Other negative regulatory molecules inhibit the inflammasome activation by targeting the K^+^ efflux (e.g., BHB), cytosolic Ca^2+^ (e.g., TMEM176B), mitochondrial function (e.g., macrophage CGI-58, IL-10, and NO).

The NLRP3 inflammasome best demonstrates the complexity of inflammasome regulation in both the priming and activation steps (Fig. 2). NLRP3 inflammasome activity is tightly regulated to prevent uncontrolled activation, since aberrant signaling is associated with various autoimmune and metabolic consequences, including exacerbation of gouty arthritis, induction of cryopyrin-associated periodic syndromes (CAPS) in susceptible individuals, and possible contribution to obesity, type 2 diabetes, and Alzheimer’s disease (AD)^14^. It is tempting to assume that the proposed link of the NLRP3 IL-1/IL-18 axis with this multitude of metabolic and degenerative diseases may hold therapeutic potential in treating these diseases, via NLRP3 inhibition. However, recent translational studies suggest a more complex disease specific function of NLRP3 inflammasome inhibition. Notably, the results of the CANTOS trial showed that, although IL-1β inhibition with canakinumab reduced cardiovascular events in patients with a history of myocardial infarction, it did not reduce the incidence of new-onset diabetes^129^.

#### Regulation in the priming step

The priming step of NLRP3 inflammasome, or “signal 1”, typically involves an NF-κB-dependent upregulation of cellular NLRP3, pro-IL-1β transcription, and de novo protein synthesis upon recognition of pro-inflammatory stimuli such as ligands for TLRs^130^. This transcription-dependent priming to activate the nuclear factor-κB is mediated by the Fas-associated death domain (FADD) and later by caspase-8^131^. Other evidence uncovered more rapid mechanisms in priming the NLRP3 inflammasome, which are transcription-independent. For example, LPS–TLR4–MyD88 signaling in macrophages can non-transcriptionally prime NLRP3 by stimulating its deubiquitination as early as after 10 min^132^. Beyond TLR4, binding of TLR3 to dsRNA promotes post-translational NLRP3 inflammasome priming through TRIF/RIPK1/FADD-dependent pathways in intermediate and late phases, both of which require the FADD/caspase-8 scaffolding function^133^. Notably, simultaneous activation of TLRs and NLRP3 by sensing various microbial ligands triggers a rapid TLR-, IRAK-1-, and IRAK-4-dependent assembly of NLRP3 inflammasome components, which can bypass the priming step and is critical for immediate innate defense^134^.

#### Post-transcriptional regulation

Emerging evidence shows that the NLRP3 inflammasome can also be regulated at the post-transcriptional level. Epigenetic factors including DNA methylation and histone acetylation have been demonstrated to regulate NLRP3 mRNA expression in response to *Mycobacterium tuberculosis* infection^135^ and bortezomib-induced painful neuropathy^136^. So far, MicroRNAs are the most studied post-transcriptional regulators of NLRP3 inflammasomes. Among these, miR-223 negatively regulates the activity of NLRP3 inflammasome by targeting the UTR-binding sites of NLRP3 in myeloid cells^137^ and was proposed to modulate innate immune responses during intestinal inflammation^138^ and DAMPs-induced acute lung injury^138^. Aberrant expression of other microRNAs also targets and inhibits the NLRP3 inflammasome in various diseased states, e.g., microRNA-7 in neuroinflammation^139^, microRNA-30e in Parkinson’s disease^140^, and microRNA-22 in coronary heart disease^141^ and oral squamous cell carcinoma^142^. More recent studies begin to unravel a previously unanticipated role of long noncoding RNAs (lncRNAs) in orchestrating inflammasome activation. Among these, antisense noncoding RNA in the INK4 locus (ANRIL) can sponge miR-122-5p to enhance NLRP3 inflammasome activation in uric acid nephropathy^143^, and MALAT1 has been implicated to sponge miR-133 in ischemia–reperfusion injured myocardium^144^. Other lncRNAs were reported to either promote or attenuate inflammasome signaling, including nuclear enriched abundant transcript 1 (Neat1)^145^ and Gm15441^146^. These promising data provide potential for miRNA- and lncRNA-targeted therapies of diseases linked to NLRP3 dysfunction. More studies are required to clarify the roles of the complex miRNA networks in fine‐tuning NLRP3 inflammasome activation to facilitate translation into clinical applications.

#### Post-translational modulation

Regulation of the NLRP3 inflammasome by post-translational modifications, including phosphorylation and deubiquitination, is increasingly studied. JNK1-mediated phosphorylation of NLRP3 at Ser194 (corresponding to human Ser198) is a key non-transcriptional priming event eliciting NLRP3 self-association and subsequent inflammasome assembly^147^. Furthermore, E3 ubiquitin ligase Pellino2 promotes the ubiquitination of NLRP3 in the non-transcriptional priming step and facilitates the activation of the NLRP3 inflammasome^148^, while FBXL2 E3 ligase-mediated ubiquitination and degradation of NLRP3 plays a negative regulatory role in this inflammasome activation^148^.

During the activation step, phosphorylation of NLRP3 by Golgi-mediated protein kinase D (PKD) at Ser293 (or human Ser295) is sufficient to trigger inflammasome assembly^149^. In contrast, PKA-induced phosphorylation of NLRP3 at mouse Ser 291 mediates negative regulation of NLRP3 inflammasome induced by bile acids^150^. Dephosphorylation of NLRP3 PYD by Phosphatase 2A (PP2A)^151^, or NLRP3 Tyr861 by protein tyrosine phosphatase non-receptor 22 (PTPN22)^152^ also promotes efficient NLRP3 activation. Besides phosphorylation, the deubiquitinating enzyme BRCA1/BRCA2-containing complex subunit 3 (BRCC3) promotes an inflammasome activation by deubiquitinating NLRP3 at its LRR domain^153^. ABRO1, a component of the BRCC3 complex, enhances NLRP3 inflammasome activation by regulating NLRP3 deubiquitination after LPS priming^154^. Other examples of ubiquitination-mediated negative regulation of the NLRP3 inflammasome include the dopamine-induced E3 ligase MARCH7^155^, Ariadne homolog 2 (ARIH2)^156^ and TRIM31^157^. Interestingly, another small molecule, nitric oxide (NO), attenuates NLRP3 inflammasome activity at the post-translational level by direct S-nitrosylation^158^.

#### Negative regulation of NLRP3 inflammasome activation

In addition to post-translational and post-transcriptional regulation, a number of molecules can directly or indirectly interact with different components of the NLRP3 inflammasome, through which they impede the assembly of NLRP3 with ASC and caspase-1. For example, the B-cell adapter for phosphoinositide 3-kinase (BCAP) interacts with the caspase-1 pseudosubstrate inhibitor Flightless-1, thereby delaying the recruitment and activation of pro-caspase-1 within the “pre-inflammasome” containing only NLRP3 and ASC, and contributing to delayed clearance of pathogens^159^. The Crohn’s disease risk factor IRGM, directly interacts with NLRP3 and ASC, blocks their oligomerization and impedes inflammasome assembly, and alternatively mediates selective autophagy of NLRP3 and ASC, thus providing protection in gut inflammatory disorders^160^. Pyrin-only protein (POP) family members including POP1^161^ and POP2^162^ block inflammasome assembly by binding to ASC and inhibiting the recruitment of ASC to NLRP3. A stress granule protein DDX3X, which is involved in both NLRP3 inflammasome and stress granules assembly, regulates NLRP3 inflammasome, thereby constituting a critical checkpoint in stressed cells^163^. Other molecules inhibiting NLRP3–ASC interaction include the orphan nuclear receptor small heterodimer partner^164^, heat shock protein 70^165^, and NLR family CARD-containing 3 (NLRC3) protein^166^.

Negative regulatory molecules targeting ion efflux, mitochondrial function, and ROS signaling likewise block NLRP3 inflammasome activation. The ketone body β-hydroxybutyrate (BHB)^167^ inhibits NLRP3 inflammasome-mediated inflammation by preventing K^+^ efflux. Transmembrane protein 176B (TMEM176B) inhibits the NLRP3 inflammasome by controlling cytosolic Ca^2+^, while targeting TMEM176B can improve antitumor activity through enhanced inflammasome activation^168^. G protein-coupled receptors, which encompass a diverse class of membrane receptors, not only initiate but also suppress NLRP3 inflammasome activation by regulating ion fluxes and mtROS production, which has been reviewed recently elsewhere^169^. NO is also regarded as a crucial negative regulator of NLRP3 inflammasome by inducing stabilization of mitochondria^158^. The inflammatory cytokine IL10 inhibits NLRP3 inflammasome activation by reducing mitochondrial ROS production^170^. Other examples of molecules inhibiting NLRP3 inflammasome activity by targeting mitochondrial function and ROS include macrophage CGI-58 modulating insulin resistance^171^, and sestrin suppressing sepsis^172^.

Negative regulation targeting other NLRP3-related signaling pathways includes, for example, myeloid β-catenin, which is required for heat shock transcription factor 1-mediated immune regulation of NLRP3 functions in murine models of hepatic ischemia/reperfusion injury^173^. Blockade of IRE1α signaling, an important pathway involved in NLRP3 inflammasome assembly, dampens its assembly and subsequent caspase-1 activation^174^.

### NAIP–NLRC4 inflammasome

The NLRC4 inflammasome is generally activated by PAMPs including bacterial flagellin and type III secretion system (T3SS) components. Unlike other NLRPs, NLRC4 partners with another NLR family member, NAIP, which directly binds to bacterial ligands. NAIPs are initially recognized and activated by PAMPs, and subsequently bind to NLRC4, releasing autoinhibition of NLRC4 and inducing inflammasome formation^175^. At the molecular level, ligand specificity is mediated by NBD-associated helical domains of mouse NAIPs^176^. A recent study revealed that caspase-7 and GSDMD are critical mediators downstream of the NAIP5/NLRC4 inflammasome for restriction of pathogenic infection^177^.

The role of post-translational modulation in regulating the activity of the NLRC4 inflammasome remains controversial. For example, phosphorylation of NLRC4 Ser533 by protein kinase Cδ (PKCδ) necessitates NLRC4 inflammasome activity against *Salmonella typhimurium* infection^178^. NLRC4 phosphorylation induced by flagellin can also prime the inflammasome for activation by NAIP5, suggesting a biphasic activation mechanism for the NLRC4 inflammasome^179^. However, another study showed that NLRC4 phosphorylation by PKCδ kinase is dispensable upon recognition of Shigella T3SS inner rod protein MxiI by NAIP2^180^. Interestingly, NLRC4 S533A interacts directly with infection-induced NLRP3, which further recruits ASC and activates caspase-1^181^. The association between NLRC4 and NLRP3 is not only noted to occur in the NLRC4 S533A mutant, but also wildtype NLRC4 and NLRP3 both can be recruited to a macromolecular complex containing ASC and caspase-1^182^. The involvement of NAIPs in NLRC4–NLRP3 interaction remains to be investigated. Besides phosphorylation, a 26S proteasome-associated protein, surg1, binds to the 91–253 residues of NLRC4 and activates NLRC4 by ubiquitination. Future studies are required to disentangle the types and sites of NLRC4 phosphorylation and ubiquitination, and the molecular mechanisms underlying positive and negative regulation of the NLRC4 inflammasome. It should be noted that regulatory mechanisms governing NAIPs are equally important. A recent study uncovered interferon regulatory factor 8 (IRF8) as a critical transcriptional regulator of different murine NAIPs, which act in concert as a prerequisite for optimal NLRC4 inflammasome activation serving defense against infectious pathogens^183^.

### NLRP6 inflammasome

NLRP6, previously called PYPAF5, has initially been identified as an activator of NF-κB and caspase-1 in different immune cells^184^. NLRP6 is characterized by high expression in murine IEC, including enterocytes^185^ and goblet cells^186^. Indeed, emerging evidence shows that NLRP6 is a central regulator of host-microbiome interactions through both inflammasome-dependent and -independent mechanisms. NLRP6 has been shown to regulate intestinal innate immune defense against enteric viral infections^187^ and diverse bacterial pathogens^188^ in an inflammasome-independent manner. Importantly, NLRP6 deficiency in IEC is associated with impaired IL-18 production^185^ and depleted caspase-1 activation^41^. The NLRP6 inflammasome in IEC has a particular importance in protection against intestinal inflammation^185^, colitis-associated tumorigenesis^189,190^ persistent enteropathogenic infection and regulation of goblet cell mucus secretion^186^. Mechanistically, commensal bacteria and microbiota-associated metabolites, such as taurine, regulate NLRP6 inflammasome signaling, IL-18 secretion, and downstream antimicrobial peptides in the intestine to shape the host–microbiome interface, via direct or indirect mechanisms not elucidated to date^41^. The NLRP6 inflammasome can also be activated by bacterial TLR ligands in specific sentinel goblet cell through TLR-Myd88 signaling, which subsequently triggers muc2 production^191^. Furthermore, lipoteichoic acid, produced by gram-positive pathogens, binds and activates the NLRP6 inflammasome through recruitment of ASC, leading to both caspase-1 and caspase-11 mediated IL-18 secretion, and ultimately exacerbating systemic infection^22^. Interestingly, stress-induced corticotropin-releasing hormone specifically inhibits NLRP6 inflammasome activation, leading to gut microbiome alterations and intestinal inflammation^192^. Future studies will need to identify more pathogenic activators and negative regulators of NLRP6 inflammasome and the mechanisms by which regulation of NLRP6 contributes to modulation of intestinal inflammasome-associated diseases.

Notably, the NLRP6 inflammasome is one the first host factors suggested to regulate the composition and function of the intestinal microbiome. Susceptibility of mice with NLRP6 deficiency to inflammation and carcinogenesis is transmissible to wild-type mice via fecal microbial transfer^185,193^. More specifically, NLRP6 has been proposed to limit intestinal colonization by specific colitogenic species. Specific-pathogen free NLRP6-IL-10 double knockout mice are prone to colitis resulting from expansion of the mucin degrading species *Akkermansia muciniphila*, mediated by the associated under-expression of IL-18^194^. This diseased phenotype is transmissible to single IL-10−/− knockout mice by oral gavage of *A. muciniphila*^194^. Importantly, these results have been shown to be vivarium and microbiome-specific while lesser or significant changes in gut microbial composition are shown to occur in mice lacking NLRP6 in some vivaria compared to their WT littermates^195,196^. Likewise, NLRP6 inflammasome baseline regulation of goblet cell function and mucus secretion appears to depend on the microbiome context, differentially driven by “sentinel goblet cell” activation by defined commensal signals^197^. Collectively, these results likely are explained by differences in the experimental design and facility-specific microbiota characteristics, leading to differential effects on inflammasome signaling^198^. Distinct community structures and, in particular, exposure to specific potentially pathogenic symbionts (pathobionts), such as members of the Helicobacteraceae family, are likely required for dysbiosis to manifest biological effects in the context of NLRP6 deficiency^199^.

### AIM2 inflammasome

The ALRs are cytosolic sensors of a variety of endogenous and exogenous ligands. AIM2 is a non-NLR protein belonging to the ALR family, whose structure is characterized by a HIN200 and an N-terminal pyrin (PYD) domains^24,200^. In a quiescent state, AIM2 is mostly expressed in the spleen, peripheral blood and the intestine, but may rapidly be induced, predominantly by type I IFN signaling upon NF-κB transcriptional activation (“signal 1”)^201^.

In eukaryotic organisms, DNA is normally absent from the cytosol. AIM2 is activated via its HIN200 domain by the cytosolic presence of dsDNA, which can be considered a danger signal^202^. AIM2 recognizes cytosolic dsDNA in a non-sequence-specific manner, however, the sequence is required to be at least 80 base pairs long^87^. ALRs bind dsDNA of multiple origins, including host, microbial, and synthetic molecules^201^. Many bacterial organisms activate AIM2, including *Francisella tularensis, L. monocytogenes, S. pneumoniae, Mycobacterium* species, *Legionella pneumophila*, and *S. aureus*^203^. Bacteriolysis is a prerequisite for pathogen recognition in order to enable cytosolic exposure of free dsDNA. Apart from bacteria, genetic materials from DNA viruses entering the cytoplasm can be recognized by AIM2. This initiates an antiviral immune response against DNA viruses such as mouse cytomegalovirus (MCMV), vaccinia virus, and human papilloma viruses^200,204,205^. However, AIM2 is not sensed by some DNA-viruses, such as human herpes simplex virus (HSV)-1^204^. This highlights that some DNA viruses may have evolved escape or inhibition strategies to this inflammasome activation. The mechanisms by which viral DNA is exposed to AIM2 in the cytosol are still unknown. Conversely, AIM2 has been demonstrated to drive anti-viral responses against some RNA viruses^206^. However, how AIM2 senses RNA remains unclear. In addition to bacteria and viruses, AIM2 participates in defense against eukaryotic pathogens such as the fungus *A. fumigatus*^207^ and the protozoan pathogen *Plasmodium berghei*^208^. Activation of the AIM2 inflammasome by dsDNA can not only mediate pyroptosis in a caspase-1-dependent manner, but also to instigate apoptosis by engaging caspase-8. The balance between pyroptosis and apoptosis depends on the amount of DNA, with pyroptosis occurring at higher transfected DNA concentrations^109^.

Beyond the recognition of foreign DNA, the sensing of self-DNA recently emerges as a crucial function of the AIM2 inflammasome. Under physiological conditions, human host DNA is compartmentalized in the nucleus and mitochondria. Alterations of the nuclear envelope integrity and subsequent release of nuclear DNA into the cytosol promote AIM2 inflammasome signaling. Perturbation of nuclear envelope integrity can therefore directly initiate an innate immune response and cell death^209^. Surprisingly, AIM2 is also shown to sense ionizing-radiation-induced DNA damage within the nucleus, thereby triggering caspase-1-mediated cell death in intestinal epithelial and bone marrow-derived cells^210^. Together, these findings highlight the regulatory functions of the AIM2 inflammasome in distressed tissues beyond its well-established role as a pathogen sensor.

**Fig. 3:**
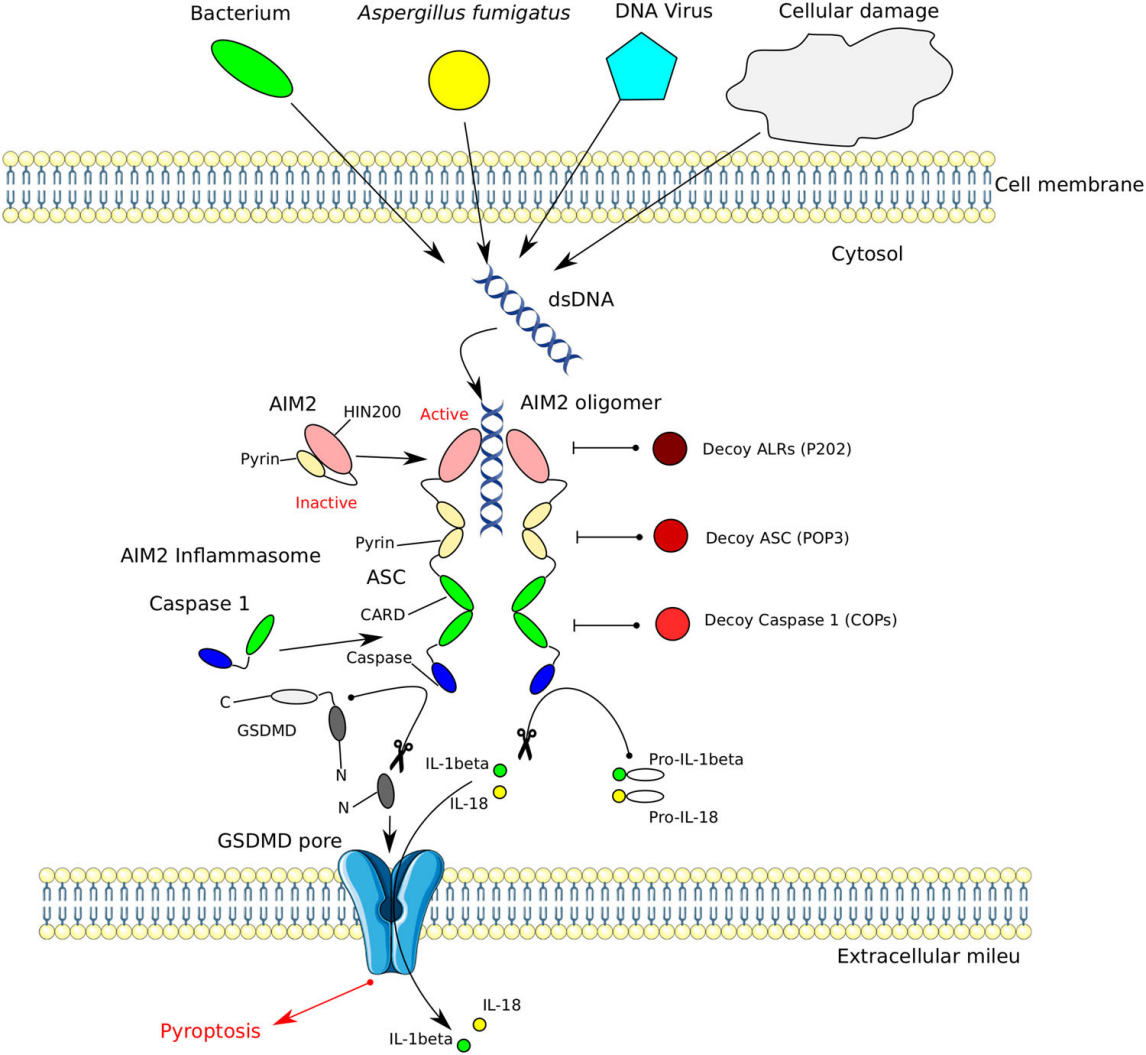
Activation and regulation of the AIM2 inflammasome. AIM2 is composed of the N-terminal pyrin (PYD) and the C-terminal HIN200 domains. Interaction of both domains renders the molecule inactive. Binding of free cytosolic double-stranded (ds)DNA releases auto-inhibition with subsequent oligomerization, recruitment of adapter ASC via PYD–PYD interaction and polymerization of the ASC protein. The dsDNA instigating the cascade can originate from microorganisms of different kingdoms and from host cellular damage. Through ASC polymerization pro-caspase-1 is recruited to the complex via CARD–CARD interactions. This induces the maturation and secretion of IL-1β and IL-18, and results in pyroptosis. AIM2 inflammasome signaling can be inhibited on several levels by decoy proteins. Decoy ALRs such as P202, which lack a PYD, may interfere with AIM2 binding to dsDNA and may also inhibit AIM2 oligomerization. POPs are decoy ASC proteins inhibit PYD–PYD interactions between ASC and AIM2. Decoy caspase-1 (COPs) lack a caspase domain and inhibit caspase-1 recruitment to the inflammasome by blocking CARD–CARD association.

Some mechanisms regulating AIM2 activity have been recently deciphered (Fig. 3). One way of regulating the AIM2 inflammasome activity involves the engagement of decoy proteins. The PYD-only proteins (POPs) represent a family of inflammasome inhibitors occupying the PYD in ASC and PYD-containing PRRs, thereby blocking PYD–PYD interactions required for inflammasome assembly^211,212^. Each main branch of inflammasome-activating PRRs in humans may have evolved a corresponding POP regulator. The type I IFN-inducible member of the POP family, POP3, was shown to inhibit the activation of ALR inflammasomes upon encounter of immunogenic DNA by interacting with PYD, thereby preventing the recruitment of ASC^213^. In addition to POPs, a protein within the ALR family has been identified as a decoy inhibitor of AIM2 in mice. Akin to AIM2, the HIN-200 protein p202 binds transfected dsDNA but lacks a PYD, therefore, preventing inflammasome activation^214^. Moreover, mouse p202 contains two HIN-domains: HIN1 attracts DNA to its surface while HIN2 mediates tetramerization of p202. The tetramers directly interact with the HIN-domain of AIM2 to prevent clustering with ASC^215^. Certain DNA sequences such as the TTAGGG repeat, commonly found in mammalian telomeres, can serve to suppress innate immune activation. A suppressive ssDNA oligodeoxynucleotide^6^ composed of four repeats of TTAGGG (ODN A151) has been shown to competitively inhibit AIM2 signaling by blocking availability of activating DNA ligands^216^. However, the in vivo role of ODNs in regulating AIM2 remains to be elucidated.

### Pyrin inflammasome

**Fig. 4:**
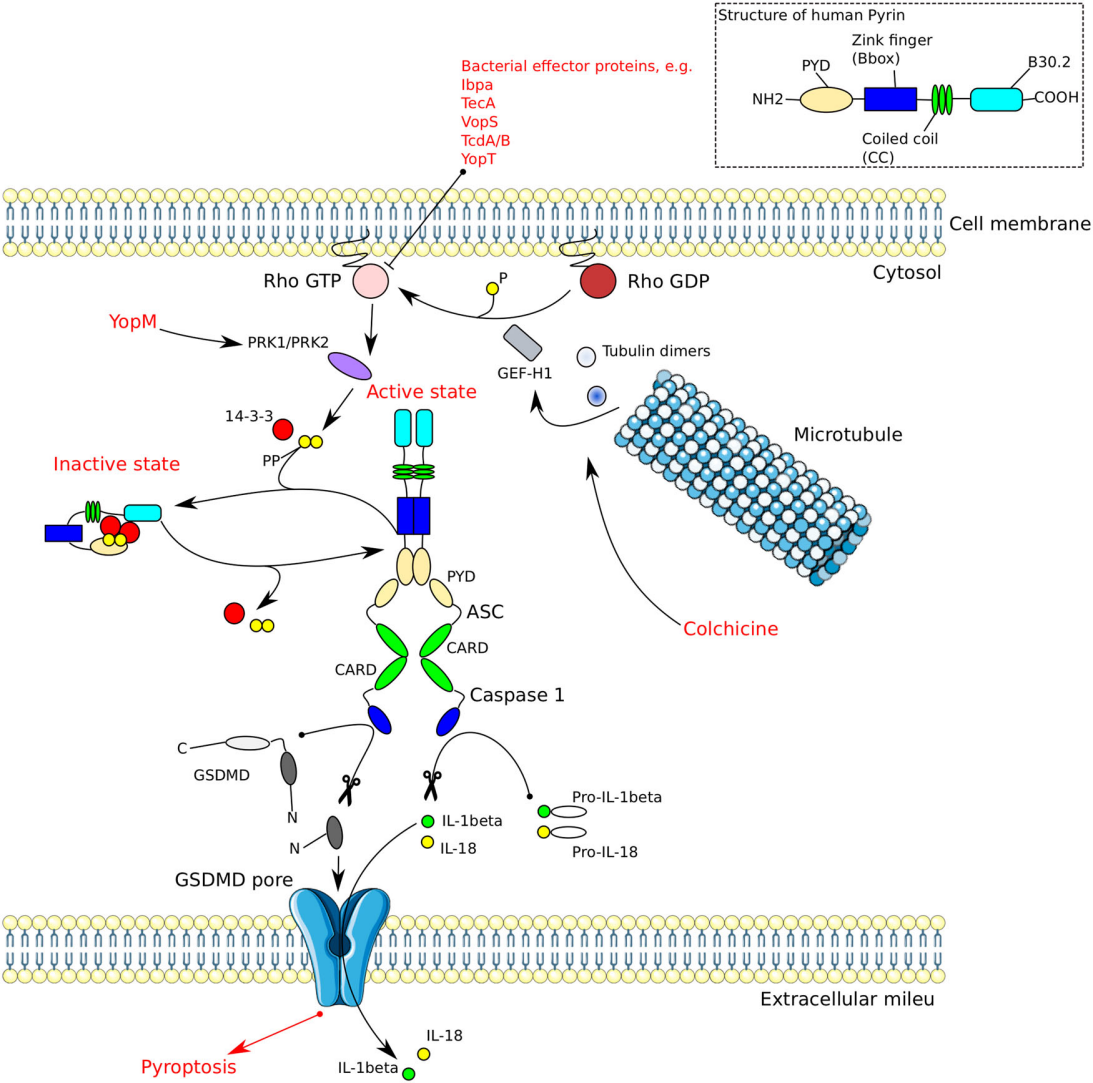
Structure, activation, and regulation of the Pyrin inflammasome. Pyrin is kept autoinhibited by 14-3-3 proteins which are bound to the phosphorylated pyrin. The inactivating pyrin phosphorylation is maintained by the kinases PRK1 and PRK2. The kinases are activated by RhoA GTPase. Inactivation of RhoA by bacterial effector proteins leads to dephosphorylation of PRK1/2. Subsequently, pyrin is de-phosphorylated, leading to dissociation of 14-3-3 and pyrin activation. Activated pyrin recruits ASC via PYD–PYD interactions. The complex then associates with pro-caspase-1 through CARD-CARD interactions. Active caspase-1 mediates IL-1β and IL-18 maturation, as well as pyroptosis. Yersinia outer protein M (YopM) suppresses the pyrin inflammasome activation by employing PRK1/2. Microtubule polymerization inhibition by colchicine leads to release of GEF-H1 which renders RhoA GTPase active, resulting in inactivating phosphorylation of pyrin by PRK1/2.

Pyrin is a high molecular mass (86 kDa) protein mostly found in immune cells such as neutrophils, monocytes and DCs^217^. Human pyrin consists of four functional domains: PYD, a zinc finger domain (bBox), a coiled coil (CC) domain, and a B30.2/SPRY domain^217^. Pyrin mediates caspase-1 inflammasome assembly in an ASC-dependent way upon recognition of an inactivating modification of the RhoA GTPase by pathogens^25,93,218^. Pyrin is a unique immune sensor, since it senses bacterial virulence via cytoskeletal remodeling rather than by microbial compounds^219^. Mouse pyrin has two functional phosphorylation sites, Ser-205 and Ser-241 (corresponding to Ser-208 and Ser 242 in humans), which render pyrin inactive by binding 14-3-3 proteins. Upon toxin stimulation or bacterial infection resulting in Rho modification, Ser-205 and Ser-241 are dephosphorylated leading to 14-3-3 dissociation. This cascade results in activation of Pyrin and formation of an oligomeric pyrin-ASC inflammasome complex (Fig. 4)^219^. Pathogenic *Yersinia spp*. secrete a virulence factor, Yersinia outer protein M (YopM), which suppresses pyrin inflammasome activation by employing host kinases PRK1 and PRK2 in order to maintain pyrin in an inactive phosphorylated state^220^. Pyrin associates with cytoskeletal microtubules and actin filaments^221^. Drugs interfering with microtubule dynamics, such as vinblastine or colchicine, inhibit pyrin inflammasome formation without affecting the pyrin phosphorylation state. This indicates that microtubule elements control the activation of the pyrin inflammasome, perhaps by impacting on the conformational change of dephosphorylated pyrin and associated ASC recruitment^219,222^. Together these concepts highlight an interesting new paradigm, in which innate immune components may engage with the cytoskeleton, thereby providing new mechanisms of structural modulation of cellular immunity.

In contrast to NLRP3 and NLRP6, knowledge regarding specific pyrin inflammasome activators produced by the residing intestinal microbiota remains scarce. In a study employing DSS-induced colitis in mice, pyrin inflammasome signaling prevented dysbiosis, promoted intestinal barrier integrity and ameliorated colonic inflammation and tumorigenesis^223^. These results shed light on an emerging role of pyrin inflammasome in maintaining intestinal homeostasis. In a recent work using whole-genome pooled CRISPR screen technology, two bile acid analogs (BAA485 and BAA473) have been identified as specific ligands inducing pyrin inflammasome signaling in myeloid and IEC lines^224^. As the enteric bacterial metagenome is a rich source of bile acid metabolism, similar microbiome-derived pyrin-inflammasome activating ligands may contribute to the regulation of intestinal homeostasis. However, the existence of such pyrin-specific ligands has not been verified in situ yet.

### Noncanonical inflammasomes

The term “non-canonical inflammasomes” was first coined in the seminal study by Kayagaki et al.^225^ to describe the finding that caspase-11 in mice (with the human orthologs caspase-4/5) is activated by mechanisms deviating from the “canonical” NLR-ASC-caspase-1 paradigm of inflammasome activation. Non-canonical inflammasome activation targets caspase-11, while loss of caspase-11 rather than caspase-1 protects mice from lethal LPS exposure^225^.

In noncanonical inflammasome signaling, caspase-11 acts as a sensor of LPS transfected into the cytosol from Gram-negative bacteria^226^. Upon recognition of LPS, caspase-11 initiates IL-1β proteolytic maturation and pyroptotic cell death in a GSDMD-dependent manner^96^. Caspase-11 directly binds to LPS via its CARD domain^227^. Noncanonical inflammasome activation, therefore, represents an LPS sensing system bypassing membrane-bound TLR4 activation and specifically defending against pathogens invading the cytosol^26^. In particular, caspase-11 is responsive to the penta- and hexa-acylated lipid A moiety of LPS, whereas tetra-acylated lipid A is not detected, providing a mechanism of evasion by the cytosol-invasive strain *Francisella spp*^228^. Caspase-11 signaling engages the NLRP3 inflammasome, thereby cross-recruiting caspase-1 signaling and eliciting maturation of IL-1β and IL-18^96^.

DCs use PRRs to sense microorganisms or dying cells in order to induce protective immunity. An oxidized phospholipid released from dying cells, oxPAPC, binds to caspase-11 in DCs to induce an inflammatory program. While binding of caspase-11 to oxPAPC and LPS signals to DCs to produce IL-1β and undergo cell death, binding to oxPAPC alone triggers DCs to secrete IL-1β and to induce a strong T-cell mediated immune response^229^. These results highlight that the outcomes of inflammasome signaling may vary depending on the biological context. Caspase-11 is regulated at both the transcriptional and posttranslational levels. Both NF-κB and signal transducer and activator of transcription (STAT) 1 bind to the promoter region of caspase-11^226^. TRIF, an adapter molecule responding to activation of TLRs, was identified as a regulator of caspase-11. TRIF is engaged by Gram-negative bacteria to activate caspase-11 via type I IFN signaling^20^. The NOD2-RIP pathway regulates ROS production and c-JUN N-terminal kinase signaling to control caspase-11 expression and subsequent activation of caspase-11 and NLRP3 inflammasome signaling during infection with Citrobacter rodentium^230^. Moreover, the complement-related Cbp1–C3a–C3aR axis increases caspase-11 expression by enhancing TLR-4–TRIF–IFNAR signaling via modulation of P38 mitogen-activated protein kinase^226^.

Similar to caspase-11 in mice, caspase-4/5 in humans are activated by binding hexa-acylated lipid A^227^. However, the biological significance and the regulation of caspase-4/5 in cytosolic recognition of LPS during bacterial infections in humans requires further investigation.

Recent studies have identified the formation of noncanonical caspase-8-dependent inflammasomes in response to different stimuli. For example, The NLRP3–ASC–caspase-8 noncanonical inflammasome can be activated upon epithelial cell apoptosis^231^, or *Cryptococcus neoformans* infection^232^, as well as by TLR stimulation in microglia^233^. Another noncanonical inflammasome complex comprising CARD9, Bcl-10, MALT1, caspase-8, and ASC is formed upon exposure to the extracellular pathogen sensor Lectin-1^234^. Besides the aforementioned caspase-8 mediated apoptosis, activation of the caspase-8-based inflammasome results in processing and maturation of IL-1β in various conditions^233,234^.

### Other inflammasomes

Much of the immunological literature is focused on the aforementioned inflammasomes. Nevertheless, the NLR and PIHYN protein families are large and entail additional molecules capable of assembling inflammasomes, including IFI16 and NLRP7. However, knowledge on their biology, regulatory mechanisms, and medical significance is still limited.

#### IFI16 inflammasome

IFI16 (interferon-inducible protein 16) is, like AIM2, a member of the PYHIN protein family, and forms an atypical inflammasome. IFI16 is composed of a PYD, HIN-A, and HIN-B domains^202^. IFI16 is present in humans, but not in mice. In contrast to its cytosolic relative AIM2, inactive IFI16 is predominantly present in the nucleus of human cells^235^. IFI16 is believed to be a “nucleus-associated inflammasome sensor component” for recognition of viruses replicating within the nucleus^235^. Upon infection with Kaposi Sarcoma herpesvirus (KSHV), IFI16, ASC, and procaspase-1 interact and are redistributed to the cytoplasm to form a functional inflammasome, leading to caspase-1 activation and IL-1β cleavage^235^. The existence of nuclear inflammasomes adds another layer of complexity to the innate intracellular immunity. Moreover, IFI16 acts as a DNA sensor that induces cell death of quiescent CD4 T cells in abortive HIV infection^236^.

#### NLRP7 inflammasome

The NLRP7 inflammasome serves multiple functions in inflammation and embryonic development^237^. NLRP7 is only present in humans and no mouse ortholog has been identified to date^238^. The NLRP7 encoding gene together with its closest relative, NLRP2, is located on the human chromosome 19. The presence of NLRP7 in various immune cells, such as macrophages, mediates the recognition of bacterial lipopeptides to promote ASC-dependent caspase-1 activation, IL-1β and IL-18 maturation, and restriction of intracellular bacterial replication^23^. Among other nonimmune tissues, NLRP7 is expressed in ovaries and oocytes and exerts its protective role in embryonic development^239^. A wide array of mutations in the NLRP7 encoding gene have been associated with manifestation of hydatidiform mole, which is a trophoblastic disease resulting in a nonviable pregnancy^237,239^. However, the mechanisms by which NLRP7 regulates embryonic development are still unclear. Beside its inflammasome forming capabilities, NLRP7 also prevents inflammasome formation^238^. These apparently contradictory findings are probably best reconciled by assuming that NLRP7 serves as a negative regulator of inflammation in quiescent cells, while in response to a proper stimulus, such as infection, NLRP7 promotes inflammasome assembly and caspase-1 activation^238^.

#### Non-inflammasome forming NLRs

As highlighted above, much of the research on NLRs focused on their function in inflammasome assembly and processing of inflammatory cytokines. However, some NLRs regulate crucial inflammasome-independent processes. Several NLR proteins, including NLRX1, NLRC3, NLRC5, and NLRP12, can negatively regulate inflammation, especially by controlling type I interferon and NFκB signaling^240–243^. Notably, NLRX1 stands out from other NLR proteins by its localization within mitochondria, where it regulates mitochondrial antiviral immunity^244,245^. Moreover, NLRC3 acts as a key inhibitory sensor of PI3K–mTOR pathways, thereby mediating protection against colorectal cancer^246^. There is no general consensus on whether NLRP12 or NLRC5 are also capable of assembling an inflammasome. Although a few studies suggest that such a putative inflammasome-dependent NLRP12 activity modulates certain infections^247^ and obesity^248^, others suggest that NLRP12’s main roles are inflammasome-independent and involve regulation of infection and colon inflammation^243,249–251^. NLRC5 has been implicated to form an inflammasome by cooperating with NLRP3 in some studies^252,253^, while others suggest that NLRC5 modulates anti-pathogenic immunity in an inflammasome-independent manner ^254,255^.

## Challenges and unknowns in inflammasome research

Despite the great advances in inflammasome research, many unknown challenging areas remain to be explored. First, although much knowledge has been gained about inflammasome activation upon bacterial pathogens, inflammasone sensing and reactivity to eukaryotic activation such as fungi, parasites and their products, awaits further investigation and discovery. Second, in addition to cytokine secretion, pyroptosis and apoptosis, the functional outputs of inflammasome activation on other cellular process, such as intestinal or skin barrier function, autophagy, mucus secretion, and more constitute attractive prospects for future research. Third, the context-specific potential for commensal microorganisms and of microbiome-associated metabolites to induce inflammasome activation needs to be further delineated in molecular detail. Given the existence of inter-facility variability in gut microbiome composition and function^198^, such regulation of inflammasome activation might be influenced by facility-dependent differences in microbiome configurations. Mechanisms mediated by commensal microbes or their products might also contribute to dampening or fine-tuning of inflammasome activation, which has yet to be determined in great details. Last, the function of many NLRs, including the NLRC, NLRP, NAIP subfamily members, remains unclarified to date. Future investigation on their inflammasome-forming ability, activation and functional consequences upon yet unknown triggers and the underlying regulating mechanisms, will greatly help to decode the complex inflammasome network and its integrative immune activities.

## Human diseases associated with inflammasome dysregulation

As highlighted above, the innate immune system is programmed to carry out a prompt and vigorous immune response towards exogenous and endogenous noxious threats. As inflammasomes represent important sensors mediating innate immune responses, it is logical that their dysregulation is associated with excessive and self-perpetuating inflammation, at different clinical contexts, which can be detrimental to the host. It is now clear that regulated or dysregulated inflammasome activation participate in most types of inflammatory disorders^256^. Inflammasome participation in the pathogenesis of multiple clinical disorders is described in more detail elsewhere^16,257,258^. In short, mutations affecting inflammasome sensor-encoding genes cause several hereditary autoinflammatory disorders, such as familial cold autoinflammatory syndrome, Muckle–Wells-syndrome or familial Mediterranean fever^259–261^. Moreover, evidence from murine and human studies associated inappropriate inflammasome activation with enhanced risk of development or progression of several common acquired human diseases, such as neurodegenerative and metabolic disorders including, among others, multiple sclerosis, AD, atherosclerosis, and type 2 diabetes^262–266^. Another rapidly advancing area of inflammasome research is their role in initiation and progression of cancer. Low-grade inflammation sustained by inflammasome singling contributes to all stages of tumorigenesis. Conversely, inflammasome activation also participates in tumor control, highlighting the diverging signals and complex roles of inflammasomes in different parts of the cancer niche^267^. The central role of inflammasomes in such a large array of diseases renders them promising drug targets, thereby meriting future basic and clinical studies.

## Recent advances in translating inflammasome research into therapeutics

To date, several inhibitors of inflammasomes have been identified and successfully implemented in disease models, with much of the research focusing on the NLRP3 inflammasome. Translation of basic inflammasome biology into therapeutic applications is a rapidly growing domain involving numerous diseases. Excellent recent reviews concisely cover this subject^267,268^. For example, sulforaphane is a naturally occurring phytochemical derived from cruciferous vegetables, such as broccoli. It inhibits caspase-1 proteolytic activation, IL-1β maturation and secretion downstream of several inflammasomes, including the NLRP1, NLRP3, and NAIP5–NLRC4 inflammasomes^269^, it and potentially also the AIM2 inflammasome^269,270^. Sulforaphane inhibits cell recruitment to the peritoneum and interleukin‐1β secretion in an in vivo model of gouty peritonitis model and reverses NLRP1‐mediated resistance to *B. anthracis* spore infection in mice^269^, as well as playing a preventive and protective role in high fat diet induced non-alcoholic steatohepatitis^271^, acute pancreatitis^272^ and diabetic retinopathy^273^. Besides, sulforaphane was suggested to be a promising drug candidate for AD therapy, as it inhibits cathepsin B- and caspase-1-dependent NLRP3 inflammasome activation induced by amyloid-β (Aβ)^274^. However, the importance of cathepsin B for crystal-induced NLRP3 responses has been debated by several groups showing normal NLRP3 activation in cathepsin B knockout macrophages^275^. Additionally, the roles of different cathepsins remain unclear due to compensatory upregulation of cathepsin activity and/or redundancy that has been suggested to account for the inability of genetic experiments to delineate a conclusive role for specific cathepsins in activating NLRP3^276^. To date, clinical studies in various inflammatory conditions utilizing sulforaphane or precursor-containing extracts have shown promising, yet inconsistent results. These have been recently reviewed elsewhere^277^. Further randomized controlled clinical studies are warranted to translate Sulforaphane use into clinical practice.

Moreover, the anti-type 2 diabetes drug glyburide, has been shown to inhibit NLRP3 inflammasome activation and consequent microbial ligand-, DAMP-, and crystal-induced IL-1β secretion^278^. Likewise, the small molecule MCC950 was demonstrated to block both canonical and noncanonical NLRP3 activation at nanomolar concentrations, to reduce IL-1β production in vivo and to attenuate the severity of experimental autoimmune encephalomyelitis (EAE) in mice, a disease model of multiple sclerosis^279^. Similarly, the small molecule CY-09 directly binds to the ATP-binding motif of NLRP3 NACHT domain and inhibits NLRP3 ATPase activity, which results in suppression of NLRP3 inflammasome assembly and activation. Administration of CY-09 showed considerable therapeutic efficacy in modulating mouse models of cryopyrin-associated autoinflammatory syndrome and type 2 diabetes^280^. Isoliquiritigenin (ILG), a flavonoid derived from the flowery plant *Glycyrrhiza uralensis*, potently inhibits LPS plus IAPP‐induced IL‐1β production in bone marrow-derived macrophages, hence improving HFD-induced obesity, lipid homeostasis, insulin resistance, and hepatic steatosis in mice^281^. Furthermore, ILG Inhibition of the NLRP3 inflammasome by triggering of Nrf2 activity was demonstrated to ameliorate LPS-induced acute lung injury^282^ and intracerebral hemorrhage in mice^283^.

Other groups of compounds shown to effectively inhibit the NLRP3 inflammasome include several clinically approved non-steroidal anti-inflammatory drugs (NSAIDs) of the fenamate class, acting via inhibition of volume-regulated anion channels in macrophages, independent of their roles on inhibiting COX enzymes^284^. The therapeutic effects of fenamates were demonstrated in models of Aβ-induced memory loss and a transgenic mouse model of alzheimer's disease^284^. Recently, microRNA-based post-transcriptional control of NLRP3 became a focus of translational research^285^. MicroRNA-9 showed promising results in inhibiting NLRP3 inflammasome activation through the JAK1/STAT signaling pathway in human cell models of atherosclerosis-associated inflammation^286^. OLT1177, a β-sulfonyl nitrile compound suggested to act as an NLRP3 inflammasome inhibitor, ameliorated metabolic perturbations associated with inflammation and proved to be safe in humans^287^. Likewise, the ketone body beta-hydroxy-butirate (BHB) or a ketogenic diet attenuated caspase-1 activation and IL-1β secretion in mouse models of NLRP3-mediated diseases such as Muckle–Wells syndrome, familial cold autoinflammatory syndrome, and urate crystal-induced peritonitis, providing a potential mechanistic explanation for the well-known anti-inflammatory effects of caloric restriction and ketogenic dieting^167^. Their therapeutic utility in patients remains to be determined.

Despite that the clinical promise is presented by the increasing number of small molecule inflammasome inhibitor candidates discovered during the past decade, their medical benefits and long-term safety still largely await proof by randomised controlled human trials. Moreover, as comparative data is limited, there is currently little evidence supporting the choice of one compound over the other in a given medical condition. Arguably, selective inflammasome inhibitors may be preferable over non-selective ones (e.g., sulforaphane), by avoiding or minimizing activation of other inflammasomes, thereby maintaining crucial functions in host defense intact while minimizing adverse effects^288^. Whether such selective and nonselective inflammasome inhibitors differ in efficacy and safety merits future studies.

## Perspectives and future directions

Recent advances in inflammasome research have greatly enhanced our understanding of how recognition of PAMPs and DMAPs by the innate immune system triggers inflammation. Given the intimate association between inflammasome dysregulation and various human diseases, it is both intriguing and challenging to unravel the complex mechanisms underlying inflammasome activation and regulation and their significance for human health and disease. First, as the structural mechanisms of canonical inflammasome assembly are progressively elucidated, future investigations will focus more on structural details regarding ligand-sensor binding, interaction between different inflammasome sensors and noncanonical inflammasome formation. Unraveling these will enable to identify how divergent signals activate inflammasomes, and how different types of inflammasomes interact with each other. Second, given the diversity of microbiome- and host-derived factors activating or inhibiting inflammasomes, particularly of the NLRP3 inflammasome, it is crucial to identify a unifying triggering pathways upstream of inflammasome activation, which will facilitate the development of potential therapeutic targets. Third, despite a growing body of research, our knowledge on molecular mechanisms orchestrating inflammasome activation remains incomplete. For example, the molecular events linking the K^+^ efflux and NLRP3–NEK7 inflammasome assembly are still elusive. Future studies are required to identify key molecules that can potently impede or promote inflammasome assembly or activation. Designing therapeutic drugs by targeting these key molecules may be a promising approach contributing to development of new disease treatments and prevention strategies. Finally, many studies focusing on inflammasome biology focused so far on small animal models. Translation to the humans is challenging, but critically necessary given the considerable interspecies divergence between murine and human inflammasomes. Therefore, it is imperative to expand future inflammasome investigations into the context of human health and disease.
